# Abundance and distribution of waterbirds in Boyo wetland, Central Rift Valley region of Ethiopia: changes in abundance and distribution

**DOI:** 10.1186/s12862-026-02579-9

**Published:** 2026-08-25

**Authors:** Hussen Yasin, Wondimagegnehu Tekalign, Serekebirhan Takele, Barry J. McMahon, Abebayehu Desalegn

**Affiliations:** 1 Department of Biology, Werabe University, P.O.Box: 46, Worabe, Ethiopia; 2 Department of Biology, Wolaita Sodo University, Sodo, Ethiopia; 3 Department of Biology, Arba Minch University, Arba Minch, Ethiopia; 4 UCD, University College Dublin, Dublin, Ireland

**Keywords:** Farmland, Natural habitat, Vulnerable species, Wetland, Wintering site

## Abstract

Waterbirds depend on wetland ecosystems. The present study aimed to investigate the species abundance and composition of waterbird species in the Boyo wetland. A bird survey was conducted from September 2022 to February 2023. There is a significance seasonal variation in waterbird distribution (Mann–Whitney U tests, *P* < 0.05). The ANOSIM test shown a significant difference in species composition and abundance of waterbirds among the study microhabitats (*R* = 0.811, *p* < 0.001). Analysis of similarity percentages also reveals dissimilarity in waterbird communities among microhabitats. Relatively higher species richness (*R* = 19) and diversity (H′ = 2.75, *J*′ = 0.93) recorded in shallow habitat. Open water habitat supports higher waterbird belong to a carnivore feeding guild. A particular importance of the wetland is the presence of the two vulnerable species, the wattled crane (Bugeranus carunculatus) and the black-crowned crane (Balearica pavonina), along with one globally threatened black-tailed godwit (Limosa limosa). Some bird species such as Wattled crane showed preference for farmland environments. This shift suggests that the destruction of natural wetland habitats may be forcing this vulnerable species to seek alternative feeding grounds. Therefore, an immediate restoration action should be taken to maintain the role of this natural wetland to serve as a wintering site for waterbird species.

**Supplementary Information:**

The online version contains supplementary material available at https://doi.org/10.1186/s12862-026-02579-9.

## Introduction

Waterbirds are among the most attractive bird species worldwide [52]. According to [62], all species in the following families are categorized as waterbirds: Podicipedidae, Pelecanidae, Phalacrocoracidae, Anhingidae, Ardeidae, Threskiornithidae, Phoenicopteridae, Anatidae, Gruidae, Rallidae, Heliornithidae, Jacanidae, Laridae, Sternidae, and skimmer. Wetlands are important to the survival of a wider variety of waterbird assemblages [10, 21]. However, wetlands have been lost or are threatened everywhere around the globe [16, 30]. The widespread degradation of wetland ecosystems has continued [22]. Over the past 20 years, human activity has jeopardized 50% of wetlands found in nature and over 17% of waterbird species worldwide [43].

Waterbirds are viewed as important bioindicators because they exhibit conspicuous and meaningful responses to changes in wetland habitats [20]. Understanding habitat selection and its effects on species distribution requires an understanding of how birds respond to the features of their local habitats and the structure of the landscape [8]. Healthy populations of wading birds serve as indicators of success in various restoration projects [5]. These species are crucial wetland system indicators because of their high mobility, adaptability in habitat selection, and quick response to habitat quality changes [4]. The significance of prey availability for wading birds and other avian predators’ ability to successfully forage has been highlighted in numerous studies [27]. For instance, wood storks and white ibises move to new feeding areas with other wading bird species as soon as prey availability starts to decrease (Jayson, 2018).

Well-managed wetlands can protect waterbirds [35]. However, the sustainability of wetland habitats is increasingly problematic because of the growing risks associated with shifting land use and climate change [17]. Competing interests in resource development and land use often conflict with wetlands [14]. Consequently, the biodiversity that natural wetlands support and the ecosystem services they offer are greatly impacted by wetland degradation [34]. Migratory networks of waterbirds are impacted [17]. Moreover, maintaining the diversity of waterbirds is essential to the healthy cycling of nutrients, storage of energy, and productivity of wetland ecosystems [59]. Determining the characteristics that put a species at risk of extinction is a basic objective of conservation biology (Carscadden et al., 2020). Thus, the conservation of waterbirds species will depend on the protection of wetlands along a spectrum of habitat attributes at different scales [19]. Therefore, it would be crucial to develop efficient wetland conservation plans by monitoring the biotic health of the wetlands [11].

Human activities in habitat of wildlife result disturbance. Thus, buffer is required between the wildlife and its observers to reduce disturbance [37]. A fundamental understanding of habitat requirements is essential for effective species management and conservation [1, 19, 32]. Our study wetland is a bird conservation area in the Central Rift Valley region. It serves as an important feeding ground for waterbirds in the region [25]. However, there are few ecological studies on the waterbird species of the area [25, 53]. Therefore, study on the abundance and distribution of waterbirds of the wetland is very crucial to know the population status and human disturbance.

## Materials and methods

### Description of the study area

**Fig. 1:**
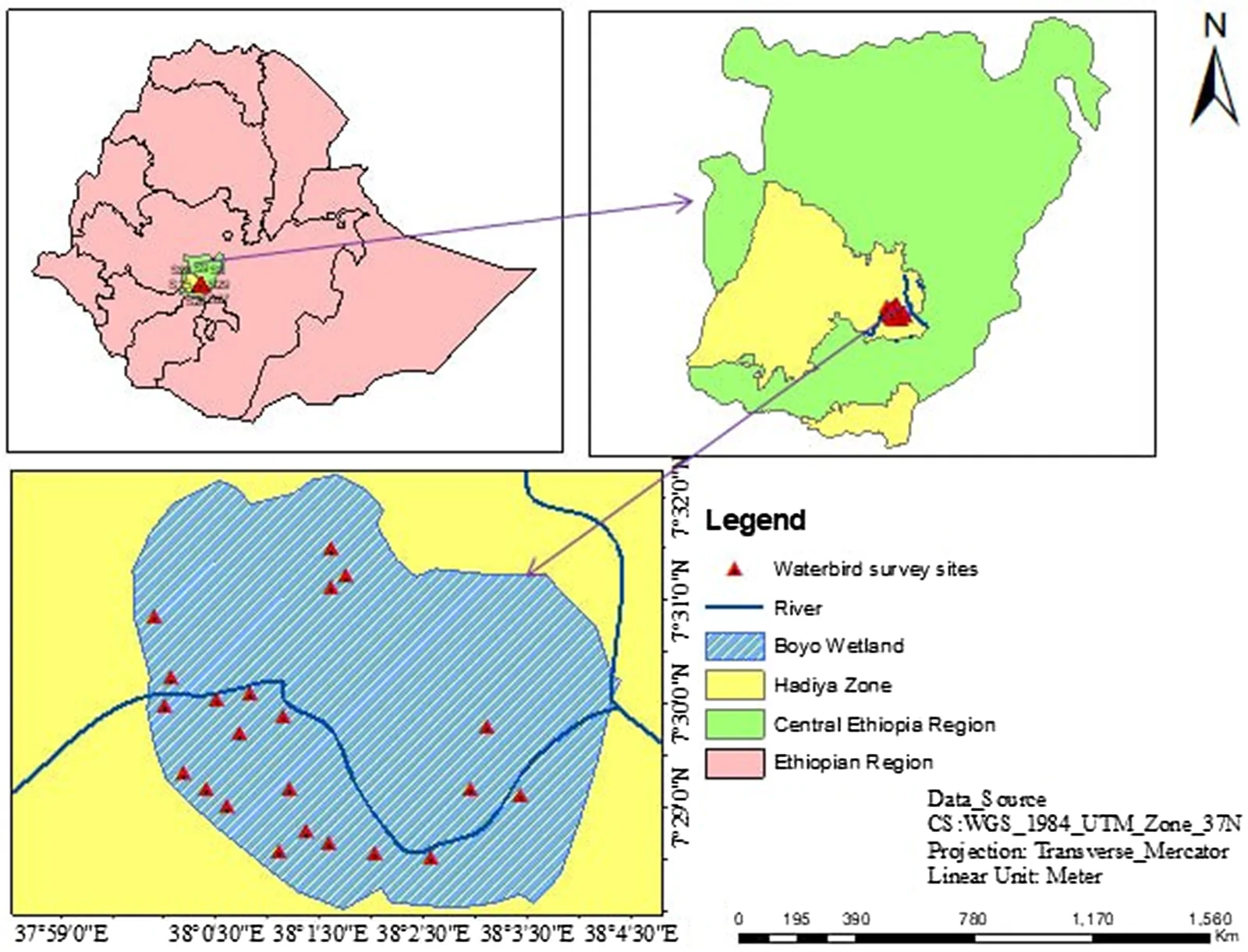
Map of the Boyo wetland with sampling sites for the present study

The study was conducted in the Boyo Wetland, which is located in the Bilate watershed, Central Rift Valley region of Ethiopia (Fig. 1). The watershed is situated at elevations ranging from 1300 m asl at Lake Abaya and 3050 m asl at Mount Ambaricho [64]. In the study area, due to its flat topography at the bottom of the watershed, the area is prone to flooding during the rainy season [31]. Concerning the vegetation cover of the area, mainly two main grass species: Cupgrass (*Eriochloa fatmensis*) and Nees pilger (*Eriochloa meyeriana*) are found in the wetland. These are important for cattle grazing while cattail (*Typha angustifolia*) is another dominant species that local people use to build huts. Ambatsch (*Aeschynomene elaphroxylon*), is the predominant bush inside the wetland and used by the locals for firewood [53]. A recent study indicated that the wetland supported 110 species of birds (both terrestrial and waterbird) across 37 families [56].

### Preliminary survey and stratification of the study area

A reconnaissance survey was conducted to gain a thorough understanding of the physical features of the study area. Based on a recent study on LULCs’ change of Boyo wetland, the study’s habitat was classified into five land use and land cover (LULCs): wetland, grazing land, barren land, farmland, and built-up areas [67]. Each habitat type inside and surrounding the wetland had sampling sites as part of the sampling strata. We divided the wetland area into two microhabitats (open and shallow water) while we merged built-up areas into farmland. So, the entire study region was divided into open water (OW), shallow water (ShW), bare land (BL), grassland (GL), and farmland (FL) near the wetland based on the observed importance as a habitat of waterbirds in the Boyo wetland (Table 1).

**Table 1:** Descriptions of the five habitat classes used for bird surveys

Habitat type	Habitat code	General description
Open water	OW	Natural or artificial bodies with standing water present most of the year
Shallow water	ShW	Wetlands characterized by standing water, typically less than 2 m
Bare land	BL	Areas characterized by minimal to no vegetation cover or less than 10% covered by plants
Grassland	GL	Land covered with grass, herbs, or shrubs used for grazing livestock
Farmland	FL	Land dedicated to the production of crops, including temporary crops (arable land)

### Waterbird survey

We carried out waterbird monthly surveys during wet (September to November) and dry season (December to February) 2022/2023. A total of 22 line transects were established, including five in the farmland habitat, six in the bare land, four in the open water area, and seven in the shallow water including the riverine habitats. A transect line of 500 m in length within a 100 m radius (except in open water) was moving steadily to scan the waterbirds [6]. Each line transect was 300 m far away from each other to avoid double count. Point counts were designed along the transect line. The survey was carried out in the early morning, from 06:30 to 08:30, and in the late afternoon, from 4:00 to 6:00. Observations were made using a binocular (8 × 40) and the “naked eye” from a fixed visible focal point for 10 min [65]. Every survey used randomization to determine the starting positions and travel directions between vantage points [55]. Once a species of bird was identified, we counted the individual birds as well as the microhabitats they were using [46]. We took photo of the bird species for further checkup to avoid misidentification. The observer walked around the wetland’s accessible edges after the point count finished for any birds that might not have been visible. All species were identified using a bird field guidebook related to the study area [26, 63].

### Data analyses

We conducted Hellinger transformation before analysis to minimize the influence of species with high abundance. To realize the conservation status of waterbirds, every taxon was compared to the [24]. We computed bird α-diversity indices, including species richness (S), the Shannon diversity index (H’), and evenness index in each site using vegan package of R. Diversity index of different species by using [49] diversity index formula:

**Figure:**
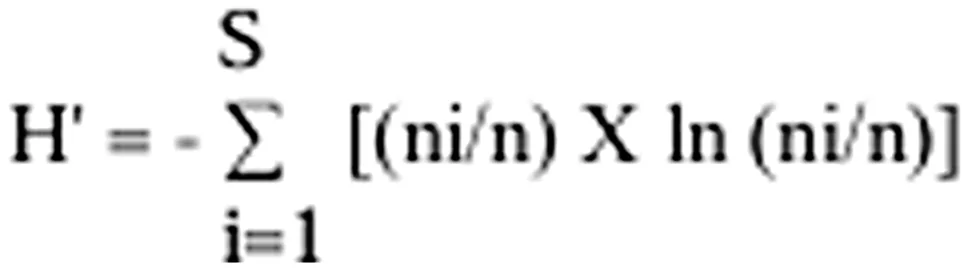

Where, ni = Number of individuals belonging to the i^th^ species.

n = Total number of individuals in the sample.

The ‘H’ index is the most commonly utilized index to describe species diversity in a community. ‘H’ gives more importance to rare species in a community. H’ varies between 0 and log2S. Higher index value represents high species diversity and clearly indicates a healthy environment [33]. Shannon’s index started performing best [42]. Evenness is complementary to the diversity index concept and it measures the evenness of species abundance and indicates how the individuals of various species are distributed in a community [23]. It ranges from zero to one. When evenness is close to zero, it indicates that most of the individuals belong to one or a few species/categories, whereas close to one indicates that each species consists of almost same number of individuals [33]. The evenness of species (E2) was estimated using the following formula:

**Figure:**
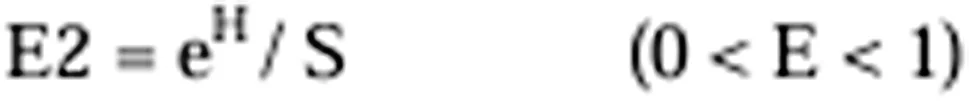

Where, e = is the natural logarithm base H’ = Shannon-Weaver diversity index S = Number of species.

To examine seasonal variation in bird community diversity in wet and dry periods, the Mann–Whitney U test was used for non-parametric comparisons between these two seasons. The analyses were conducted using the wilcox.test () functions in R 4.5.3, ensuring robustness against non-normal distributions [68].

Multivariate analysis tests, including Analysis of Similarities (ANOSIM) and Similarity Percentages (SIMPER), were employed to analyze β diversity to describe community variations. The statistical significance of the differences among habitat types based on species abundance was tested using ANOSIM [13]. Since the ANOSIM procedure does not assume anything about the data in advance, unlike ANOVA, it is more appropriate for community data [50]. The value of the R statistic in ANOSIM measures directly the degree of distinctiveness of groups, regardless of sample size. R ranges from − 1 to 1, negative values of R indicate that the average of the ranks of within-group dissimilarities is greater than the average of the ranks of between-group dissimilarities [2]. We used the Bray–Curtis similarity index to create a dissimilarity matrix between sites and a dendrogram was employed to display the species composition and abundance across microhabitats using Euclidean distance [39]. The required feeding guild data of the waterbirds was classified into four primary feeding guilds based on existing literature and similarities in their feeding ecology [57]. The analysis was done with R (R core team, software version 4.5.3).

## Results

### Composition and relative abundance of the waterbird community in the study area

**Fig. 2:**
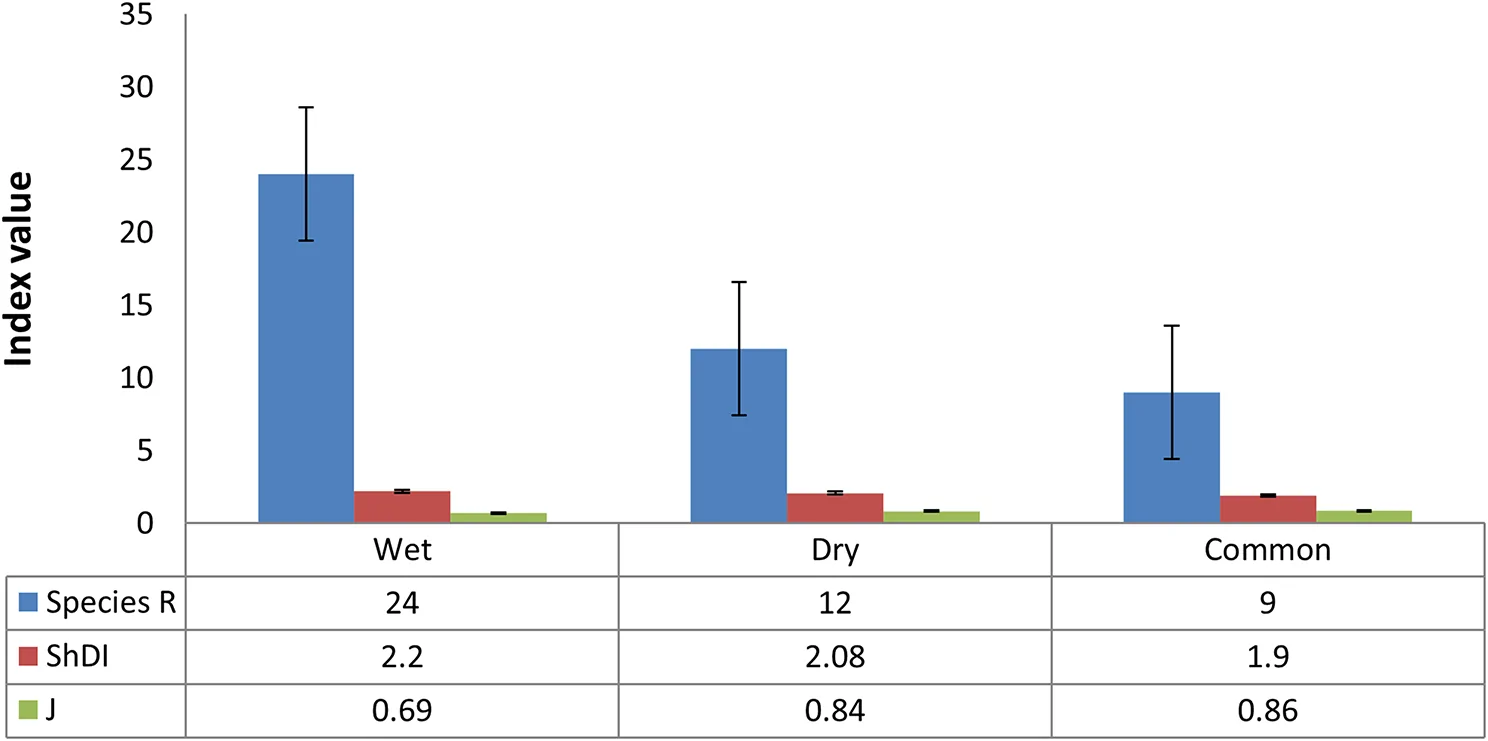
Diversity indices of waterbird recorded in different season (Error bar= standard error)

There is a significant seasonal variation in waterbird abundance and distribution (Mann–Whitney U tests, *P* < 0.05). In wet season, both diversity (*H*′ =2.2) and species richness (*R* = 24) relatively higher than from dry season record (*H*′ =2.08, *R* = 12). Nine species are common in both seasons (Fig. 2). Order Pelecaniformes had ten species, followed by Charadriiformes with seven. The three dominant waterbird species are Common crane (*Grus grus)*, Egyptian goose (*Alopochen aegyptiaca*) and Cattle egret (*Bubulcus ibis)*. The majority of the recorded waterbirds are waders while some are waterfowl. According to the IUCN Red List (2025), the Wattled crane and the Black-crowned crane are vulnerable species while the Black-tailed godwit is near-threatened species (Appendix).

## Habitat utilization and overlap of waterbirds in the study area

**Fig. 3:**
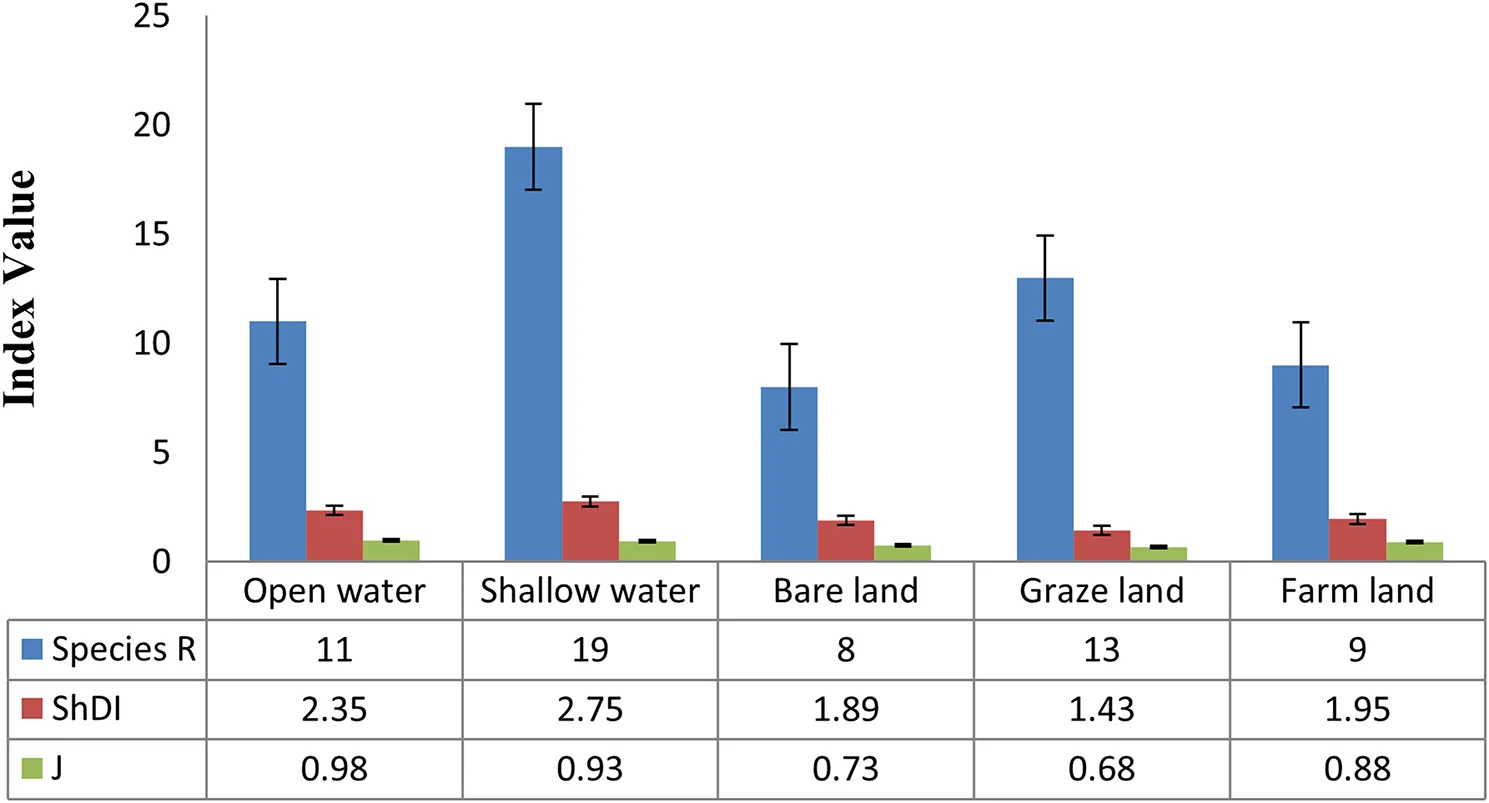
Diversity indices of waterbird recorded in different habitats (Error bar= standard error). Note: (J′= evenness index, ShDI= Shannon diversity index (H′), Species R= species richness)

As observed in the bar graph below, the majority of waterbirds recorded in shallow water habitat. The highest species richness and diversity of waterbirds in the study area (*R* = 19 and *H*′ = 2.75, respectively) were recorded in this habitat. The open water was found to be the second highest (*H*′ = 2.75). Relatively, the lowest diversity (*H*′ = 1.43) was recorded in the grassland (Fig. 3).

**Fig. 4:**
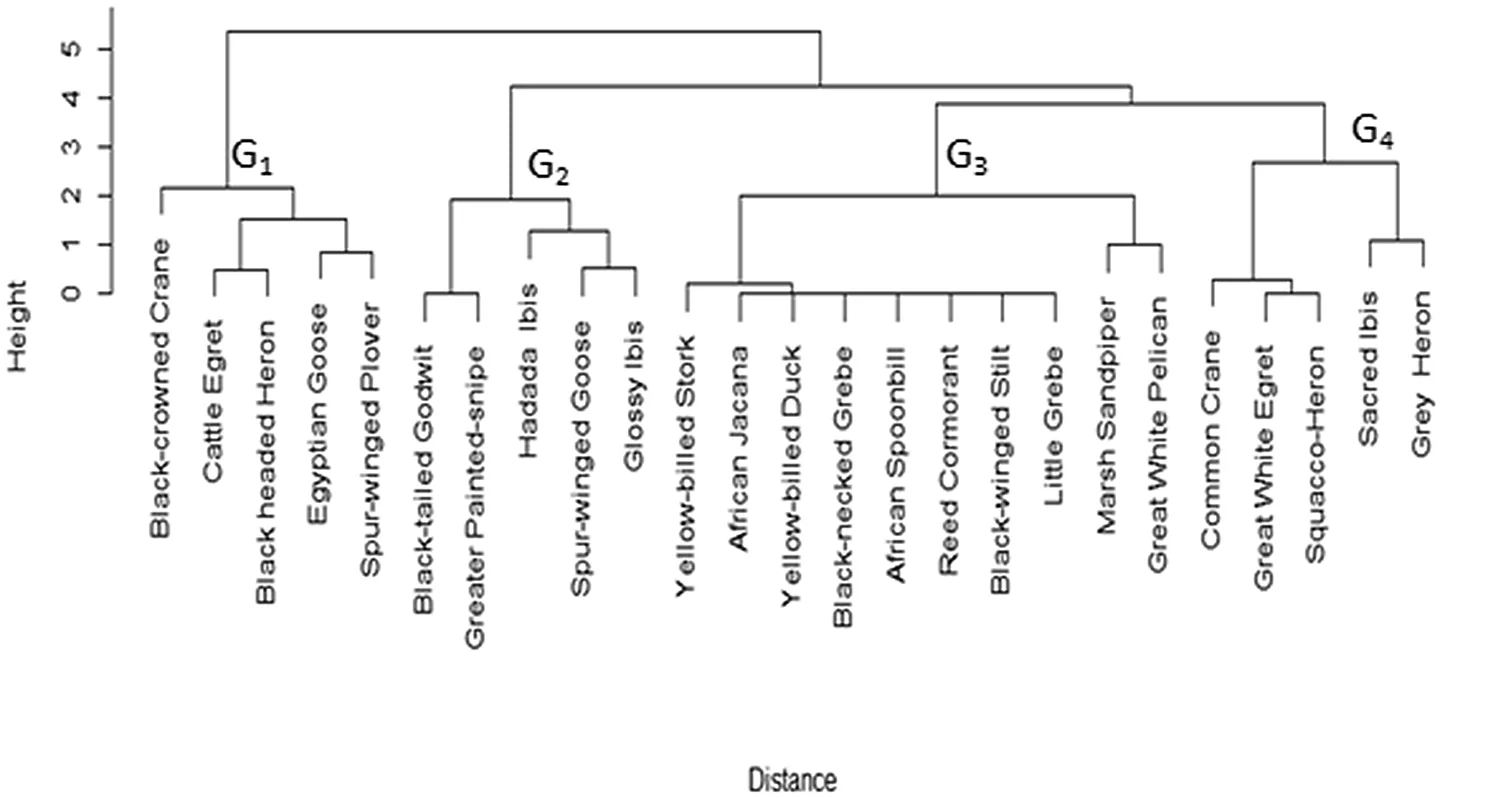
Waterbird species clustering based on similarities in habitat use by using average Euclidean distances (G stands for group that represents clustering)

The dendrogram shows the overlap of waterbirds species clustering based on similarity in habitat utilization. Group one (G1) comprises five species: Black-crowned Crane, Cattle Egret, Black-headed Heron, Egyptian Goose, and Spur-winged Plover. Group two (G2) comprises five species: Black-tailed Godwit, Greater Painted-snipe, Hadada Ibis, Spur-winged Goose and Glossy Ibis. Group three (G3) comprises ten species: Yellow-billed Stork, African Jacana, Yellow-billed Duck, Black-necked Grebe, African Spoonbill, Black-winged Stilt, Reed Cormorant, Little Grebe, Marsh Sandpiper and Great White Pelican. Five species make up the fourth group (G4): Common Crane, Great White Egret, Squacco Heron, Sacred Ibis and Grey Heron (Fig. 4).

### Waterbird community variation in habitat-wise comparison

There is a significant differences in habitat preference among waterbird species recorded in the five microhabitats, as indicated by a one-way ANOSIM using the Bray-Curtis similarity index (*R* = 0.811, *p* < 0.001). According to analysis of similarity percentages (SIMPER) result, the dissimilarity between OW and ShW was primarily linked to Common crane, Egyptian goose and Yellow billed stork with overall dissimilarity 95.74% at a significant level (*p* < 0.05). The dissimilarity between OW and GL was mainly linked with Yellow billed Stork, Egyptian goose and Common crane with overall dissimilarity 93.02% (*p* < 0.05). The variation between OW and BL was mainly due to Yellow billed stork, Egyptian goose and Back-necked grebe with a 88.83% dissimilarity (*p* < 0.05). The variation within these two pairs of habitats (ShW vs. FL and ShW vs. GL) was mainly due to Common crane, Egyptian goose and Yellow billed stork with a dissimilarity level (91.38% and 90.26%, respectively) was significance (*p* < 0.05). The dissimilarity within these three pairs of habitat types (i.e. GL vs. BL, GL vs. FL, and FL vs. BL) was not significance (*p* > 0.05) (Table 2).

**Table 2:** SIMPER and ANOSIM analysis results of pairwise comparison of waterbird communities between different habitat types in study area

Habitat Type	SIMPER				ANOSIM	
	Most discriminating species	Contrib (%)	Cum.(%)	Overall Av. Dissimilarity (%)	R	P
OW vs. ShW	Common crane	78.38	78.38	95.74	1	**0.032**
	Egyptian goose	11.28	89.66			
	Yellow billed stork	2.34	92.01			
OW vs. GL	Yellow billed Stork	45.53	45.53	93.02	1	**0.028**
	Egyptian goose	31.25	76.78			
	Common crane	5.16	81.95			
OW vs. BL	Yellow billed stork	65.53	65.53	88.83	0.395	**0.027**
	Egyptian goose	15.98	81.51			
	Back –necked grebe	5.98	87.49			
ShW vs. FL	Common crane	86.44	86.44	91.38	1	**0.029**
	Egyptian goose	4.20	90.64			
	Yellow billed stork	1.85	92.5			
ShW vs. GL	Common crane	84.5	84.5	90.26	1	**0.028**
	Egyptian goose	6.54	91.05			
	Yellow billed stork	1.8	92.89			
GL vs. BL	Egyptian goose	59.29	59.29	61.89	0.15	0.1
	Common crane	12.96	72.24			
	Wattled crane	8.24	80.49			
GL vs. FL	Egyptian goose	65.18	65.18	46.52	0	0.51
	Wattled crane	11.31	76.49			
	Common crane	6.19	82.69			
FL vs. BL	Egyptian goose	43.22	43.22	64.49	0.02	0.33
	Wattled crane	20.04	63.26			
	Common crane	11.21	74.47			

(ShW: Shallow water, OW: Open water, GL: Grass land, FL: Farm land, and BL: Bare land) in the BW)

Note: the average dissimilarity is the contribution of each species to the dissimilarity between the group pairs according to Bray-Curtis similarity. Only three species that contributed the most to the dissimilarity of habitat are considered. Contribution (Contrib (%) indicated the percentage contribution of each species to the group pair dissimilarity in percentage values. Cumulative **(**Cum.%) indicates the percentage cumulative value of the contribution of each species to the dissimilarity. Overall Av. dissimilarity, quantifies the total percentage of difference (0–100) between groups, R-value (R- Global), P-value -Significant value (Bold p-value indicate significant at 0.05)

**Fig. 5:**
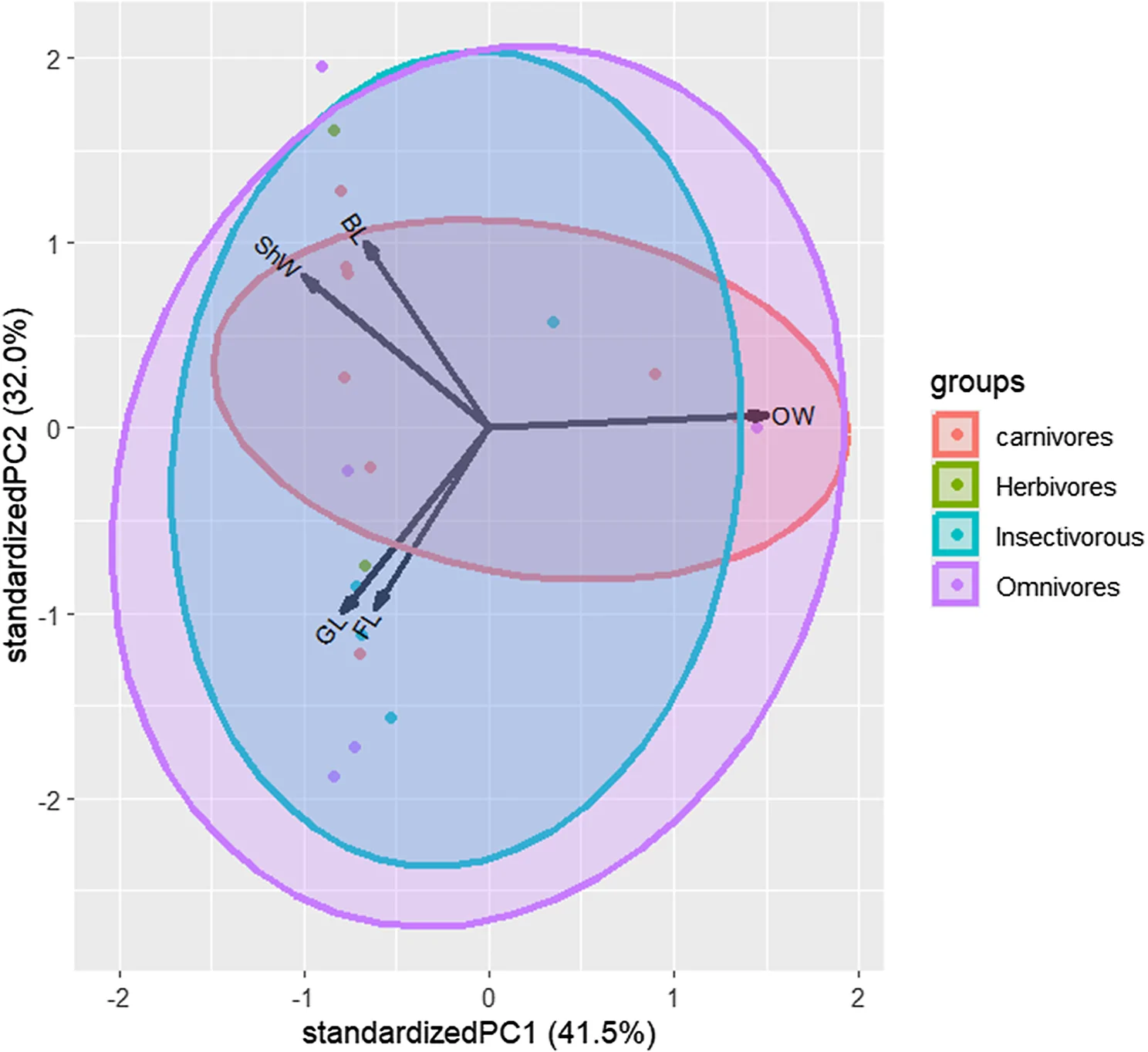
The clustering of habitat types based on the similarity in waterbirds’ feeding guild. (ShW: Shallow water, OW: Open water, GL: Grass land, FL: Farm land, BL: Bare land)

Shallow water and bare land show closer similarity based on feeding guild of waterbird and grassland and farmland also show similarity. Open water habitat is particularly support distinct feeding guild with no similarity with other habitat types. Mostly carnivore feeding group recorded in open water habitat (Fig. 5).

## Discussion

As the study reveals that higher waterbird species was recorded in wet season. In this season, availability of food after a seasonal flood might attract bird species. Waterbirds react directly to increased food supplies in nearby basins after flooding [60]. The existence of food resources, such as fish, amphibians, reptiles, invertebrates and vegetable matter, is a significant factor that determines the habitat’s suitability and affects the reproductive success of wetland birds [44]. Flooding is necessary for the diversity and waterbirds in floodplain wetlands. Total bird diversity and abundance may be most likely to be enhanced by variations in the flooding regime at wetlands [9]. Even within the same general vegetation type, changes in the frequency of flooding are linked to substantial changes in the site’s character and, eventually, changes in the makeup of the bird population [36]. Therefore, the regional variability of bird communities is significantly influenced by flood seasonality, with the wettest phase exhibiting the greatest differences in species composition [15].

Over 60% waterbird species recorded in this study preferred shallow water. The observed high diversity may be attributed to the suitability of the habitat and the availability of food resources. Factors such as food supply, adequate shelter, and suitable breeding habitats play a crucial role in influencing species richness and abundance [38]. Waterbirds prefer mudflats and shallow water habitats [29, 32]. Compared to manmade wetlands, natural wetlands support a wider variety of waterbird assemblages, making them an essential focus of protection concerns [43]. The decline and loss of wetlands habitats have negatively impacted waterbird populations [47]. Therefore, providing habitat for waterbirds whose survival depends on wetlands should be a primary objective of many wetland restoration programs [21].

The study revealed that in the microhabitats with high human disturbance (such as farmland), less species diversity was recorded. The rising anthropogenic activities (mainly agriculture and cattle grazing ) have put the diversity of waterbirds at risk [12]. Many other anthropogenic impacts such as sand mining, waste disposal, and development disturbance might affect the study wetland [66, 67]. If these pressures persist, shorebird assemblages may continue to decline [40, 48]. Hence, the habitats of waterbirds are at risk of fragmentation and loss as a result of the prolonged negative impact of human activity, which presents a significant threat to their survival and diversity [51]. A previous study in the study area indicates that the Wattled crane, a vulnerable bird species, spends more time foraging on farmland than in wetlands, which might suggest that the amount of food in their primary natural habitat may have decreased [54]. As natural wetlands are gradually disappearing due to human activity, species that depend on water are finding new habitats. A significant number of waterbirds worldwide are seeking sanctuary in agricultural wetlands [17, 18]. Therefore, the protection of avian species through the provision of undisturbed roosting and breeding sites requires a minimized anthropogenic stressors [46].

Disturbance frequencies may have a significant effect on waterbirds and may have an impact on population size [45]. The restoration of wetland habitats and the improvement of natural food sources are crucial for waterbirds [54]. Wetland restoration is essential for preserving biodiversity, safeguarding ecosystem services, and avoiding habitat degradation [41, 28]. Restoring is an approach to improve the ecological suitability for waterbirds [3]. To reduce the effects of human disturbance, protected areas must be designated around natural wetland [7]. Because protecting waterbirds is a complex issue, cooperation between ornithologists, hydrologists, politicians, and residents is needed [61]. Therefore, encouraging the implementation of conservation programs and improved intergovernmental cooperation are critical to the success of the global effort to conserve migratory bird species [52, 58].

## Conclusion

The finding confirms that Boyo wetland is a crucial habitat for a diverse range of waterbird species, including the common crane, Egyptian goose, Great white pelican, African spoonbill, and several ibis and egret species. Of the particular importance this wetland is the presence of the two vulnerable species, the wattled crane and the black-crowned crane, along with one globally threatened black-tailed godwit. While most waterbirds recorded prefer shallow water microhabitats, species like the Wattled crane showed a stronger preference for farmland environments. This shift suggests that the destruction of natural wetland habitats may be forcing this vulnerable species to seek alternative feeding grounds. To ensure the conservation of waterbirds in the study area buffer zone establishment around the wetland could minimize the waterbird disturbance from human activities. Therefore, the wetland should be protected from direct human exposure.

## Supplementary Information

Below is the link to the electronic supplementary material.


Supplementary Material 1


## Data Availability

Data will available upon request.
