# Supplementary material for: Abundance and distribution of waterbirds in Boyo wetland, Central Rift Valley region of Ethiopia: changes in abundance and distribution
Source: BMC Ecol Evol. 2026 Aug 25;26:74. doi: 10.1186/s12862-026-02579-9 (PMC13528106; doi:10.1186/s12862-026-02579-9)
Supplement: Supplementary file 1 [file 12862_2026_2579_MOESM1_ESM.docx]

**Appendix**

Table S1. List of waterbird species recorded in the study area with individual counts, their respective feeding guild, migratory status, and IUCN Red List rank in 2025

| Order (Family) | Common Name | Scientific Name | #Count (RA) | Feeding Guild | Migration Status | | IUCN Red Lists Status (2025) |
| --- | --- | --- | --- | --- | --- | --- | --- |
| Ciconiiformes  (Ciconiidae) | Yellow-billed Stork | Mycteria ibis | 150(2.25) | Carn. | RB | | LC(↓) |
| Suliformes (Phalacrocoracida) | Long tailed Cormont | *Phalacrocorx africanus* | 1(0.18) | Carn. | RB | | LC(↓) |
| Gruiformes (Gruidae) | Black-crowned Crane | *Balearica pavonina* | 3(0.32) | Omn. | RB | | VU(↓) |
|  | Common Crane | *Grus grus* | 1770(7.72) | Omn. | PW | | LC(↑) |
|  | Wattled Crane | *Bugeranus carunculatus* | 89(1.73) | Omn. | PW | | VU(↓) |
| Anseriformes |  | | | | | | |
| (Anatidae) | Egyptian Goose | *Alopochen aegyptiaca* | 527(4.21) | Herb. | RB | | LC(↓) |
|  | Spur-Winged Goose | *Plectropterus gambensis* | 12(0.64) | Herb. | RB | | LC(↑) |
|  | Yellow-billed Duck | *Anas undulats* | 9(0.55) | Omn. | RB | | LC(St) |
| Podicipediformes |  | | | | | | |
| (Podicipedidae) | Little Grebe | *Tachybaptus ruficollis* | 3(0.32) | Carn. | RB | | LC(↓) |
|  | Black-necked Grebe | *Podiceps nigricollis* | 49(1.28) | Carn. | RB | | LC(St) |
|  |  | | | | | | |
| Charadriiformes |  | | | | | | |
| (Charadriidae) | Spur-winged Plover | *Vanellus spinosus* | 44(1.22) | Insec. | RB | | LC(↑) |
|  | Crowned Lapwing | *Vanellus coronatus* | 2(0.26) | Insec. | RB | | LC(St) |
| (Jacanidae) | African Jacana | *Actophilornis africanus* | 1(0.18) | Omn. | RB | | LC(St) |
| (Scolopacidae) | Black-tailed Godwit | *Limosa limosa* | 2(0.26) | Insec. | PW | | NT(↓) |
|  | Marsh Sandpiper | *Tringa stagnatilis* | 3(0.32) | Insec. | PW | | LC(↑) |
|  | Greater Painted- snipe | *Rostratula benghalensis* | 1(0.18) | Omn. | RB | | LC(↓) |
| (Recurvirostridae) | Black-winged Stilt | *Himanotopus himanotopus* | (0.26) | Insec. | RB/PW | | LC(↑) |
| Pelecaniformes |  | | | | | | |
| (Threskiornithidae) | Hadada Ibis | *Bostrychia hagedash* | 3(0.32) | Carn. | RB | LC(↑) | |
|  | Sacred Ibis | *Threskiornis aethiopicus* | 31(1.02) | Carn. | RB | LC(St) | |
|  | Glossy Ibis | *Plegadis falcinellus* | 10(0.58) | Carn. | PW/AM | LC(↓) | |
|  | African Spoonbill | *Platalea alba* | 2(0.26) | Carn. | RB | LC(St) | |
| (Ardeidae) | Cattle Egret | *Bubulcus ibis* | 183(2.48) | Insec. | RB/M | LC(↑) | |
|  | Great White Egret | *Ardea alba* | 4(0.37) | Carn. | R/PW | LC(Un) | |
|  | Black-headed Heron | *Ardea melanocephala* | 3(0.32) | Carn. | RB | LC(↑) | |
|  | Grey Heron | *Ardea cinerea* | 14(0.69) | Carn. | R/PW | LC(Un) | |
|  | Squacco-Heron | *Ardeola ralloides* | 3(0.32) | Carn. | RB/PW | LC(Un) | |
| (Pelecanidae) | Great White Pelican | *Pelecanus onocrotalus* | 51(1.31) | Carn. | RB | LC(Un) | |

Carn: Carnivores, Insec: Insectivores, Omn: Omnivores, Herb: Herbivores, RA: relative abundance , RB/PWV: there is both a resident breeding population and a wintering population; LC: least concerned; VU: vulnerable; NT: near threatened; PW: breeds in the Palearctic and winters in the country; AM: intra-Africa
